# Comparative genomic analysis identifies potential adaptive variation in Mycoplasma ovipneumoniae

**DOI:** 10.1099/mgen.0.001279

**Published:** 2024-08-30

**Authors:** Kimberly R. Andrews, Thomas E. Besser, Thibault Stalder, Eva M. Top, Katherine N. Baker, Matthew W. Fagnan, Daniel D. New, G. Maria Schneider, Alexandra Gal, Rebecca Andrews-Dickert, Samuel S. Hunter, Kimberlee B. Beckmen, Lauren Christensen, Anne Justice-Allen, Denise Konetchy, Chadwick P. Lehman, Kezia Manlove, Hollie Miyasaki, Todd Nordeen, Annette Roug, E. Frances Cassirer

**Affiliations:** 1 Institute for Interdisciplinary Data Sciences, University of Idaho, Moscow, ID, USA; 2 Department of Veterinary Microbiology and Pathology, Washington State University College of Veterinary Medicine, Pullman, WA, USA; 3 Department of Biological Sciences, University of Idaho, Moscow, ID, USA; 4 Department of Physiology and Pharmacology, College of Osteopathic Medicine, Sam Houston State University, Conroe, TX, USA; 5 Alaska Department of Fish and Game, Fairbanks, AK, USA; 6 Department of Animal, Veterinary and Food Sciences, University of Idaho, Moscow ID, USA; 7 Arizona Game and Fish Department, Phoenix AZ, USA; 8 South Dakota Department of Game, Fish and Parks, Custer, SD, USA; 9 Department of Wildland Resources and Ecology Center, Utah State University, Logan, UT, USA; 10 Idaho Department of Fish and Game, Idaho Falls, ID, USA; 11 Nebraska Game and Parks Commission, Alliance, NE, USA; 12 Utah Division of Wildlife Resources, Salt Lake City, UT, USA; 13 Idaho Department of Fish and Game, Lewiston, ID, USA

**Keywords:** *Capra hircus*, *dN/dS*, homologous recombination, *Ovis aries*, *Ovis canadensis*, pangenome

## Abstract

*Mycoplasma ovipneumoniae* is associated with respiratory disease in wild and domestic Caprinae globally, with wide variation in disease outcomes within and between host species. To gain insight into phylogenetic structure and mechanisms of pathogenicity for this bacterial species, we compared *M. ovipneumoniae* genomes for 99 samples from 6 countries (Australia, Bosnia and Herzegovina, Brazil, China, France and USA) and 4 host species (domestic sheep, domestic goats, bighorn sheep and caribou). Core genome sequences of *M. ovipneumoniae* assemblies from domestic sheep and goats fell into two well-supported phylogenetic clades that are divergent enough to be considered different bacterial species, consistent with each of these two clades having an evolutionary origin in separate host species. Genome assemblies from bighorn sheep and caribou also fell within these two clades, indicating multiple spillover events, most commonly from domestic sheep. Pangenome analysis indicated a high percentage (91.4 %) of accessory genes (i.e. genes found only in a subset of assemblies) compared to core genes (i.e. genes found in all assemblies), potentially indicating a propensity for this pathogen to adapt to within-host conditions. In addition, many genes related to carbon metabolism, which is a virulence factor for Mycoplasmas, showed evidence for homologous recombination, a potential signature of adaptation. The presence or absence of annotated genes was very similar between sheep and goat clades, with only two annotated genes significantly clade-associated. However, three *M. ovipneumoniae* genome assemblies from asymptomatic caribou in Alaska formed a highly divergent subclade within the sheep clade that lacked 23 annotated genes compared to other assemblies, and many of these genes had functions related to carbon metabolism. Overall, our results suggest that adaptation of *M. ovipneumoniae* has involved evolution of carbon metabolism pathways and virulence mechanisms related to those pathways. The genes involved in these pathways, along with other genes identified as potentially involved in virulence in this study, are potential targets for future investigation into a possible genomic basis for the high variation observed in disease outcomes within and between wild and domestic host species.

## Data Summary

Raw sequence data and genome assemblies generated for this study have been deposited with the National Center for Biotechnology Information (NCBI) under BioProject number PRJNA1070810. Assemblies are also available for download through Dryad at https://doi.org/10.5061/dryad.ffbg79d2h.

NCBI accession numbers and associated metadata for each assembly are available in the Supplemental Materials. DNA sequences extracted from these assemblies for four genetic markers [gyrase B (gyrB), RNA polymerase B (rpoB), 16S rRNA, and the 16S-23S intergenic spacer region (IGS)] are available in the Supplemental Materials. Analysis code is available at https://github.com/kimandrews/Movi, and an interactive phylogeny is available at https://nextstrain.org/community/narratives/kimandrews/Movi.

**Impact Statement**

*Mycoplasma ovipneumoniae* causes respiratory disease in wild and domestic sheep and goats around the world, resulting in economic losses for the domestic sheep industry and severe population declines for wild species. Disease outcomes vary widely within and between host species, and this variation could be influenced by genomic differences across bacterial strains. We compared *M. ovipneumoniae* genomes from six countries and four host species and found species-level divergence for strains from domestic goats versus domestic sheep, indicating separate evolutionary origins in these two host species. All wildlife strains fell within these two groups, providing evidence that these strains originated by transmission from domestic populations. We identified genes potentially involved in adaptation to hosts, which could be responsible for differences in disease outcomes across bacterial strains and host species. Many of these genes had functions related to carbon metabolism, a potential virulence factor for Mycoplasmas.

## Introduction

*Mycoplasma ovipneumoniae* (also referred to as *Mesomycoplasma ovipneumoniae* [1,2]) is a common cause of respiratory disease in domestic sheep (*Ovis aries*) and goats (*Capra hircus*) across the globe and may be increasing in prevalence in many countries [3,5]. Although *M. ovipneumoniae* infection does not typically cause high mortality rates in domestic flocks, it can significantly decrease lamb production, resulting in a cumulative economic burden [6,9]. This pathogen has also been reported in wild Caprinae species and occasionally other free-ranging and captive ungulates, likely resulting from spillover from domestic sheep and goats [5,10,12]. In contrast to the relatively low mortality rates caused by *M. ovipneumoniae* in domestic species, infection with this pathogen has been associated with severe pneumoniae in wildlife species including many populations of bighorn sheep (*Ovis canadensis*), free-ranging Norwegian muskox (*Ovibos moschatus*) and a population of captive Dall’s sheep (*Ovis dalli dalli*) [12,15]. Furthermore, *M. ovipneumoniae* infection can impose long-term constraints on population growth in wild bighorn sheep populations, likely due to transmission from chronic adult carriers to neonates [15,18]. However, disease outcomes can vary widely both within and between host species [19,22], and the factors driving this variation remain unknown. Genetically based differences in pathogenicity mechanisms across strains may be a factor contributing to this variation, along with other factors related to the host immune system or environmental conditions. Mechanisms of pathogenicity are poorly understood for *M. ovipneumoniae*, but several virulence factors have been proposed, including the presence of a polysaccharide capsule, production of hydrogen peroxide as a by-product of metabolism of the carbon-containing molecule glycerol, secretion of hemolysins and invasion of host cells to evade the host immune response [23,27].

Comparative genomics is a powerful tool that can help uncover mechanisms of pathogenicity and identify potential gene targets for assessing the role of genomic variation in disease outcomes. This approach involves sequencing entire bacterial genomes and then comparing genomic composition across strains and species. For bacterial species with unknown virulence mechanisms, this approach can be used to determine whether genes are present that are known to be virulence factors in other, more well-studied species (e.g. [23,28, 29]). This approach can also be used to identify genomic variation potentially responsible for differences in pathogenicity observed across closely related strains or species. For example, comparison of the genomes of pathogenic versus nonpathogenic strains has been used to identify genes that may be virulence factors for *Mycoplasma* species infecting domestic pigs, crocodilians and birds [30,33]. Similarly, analyses comparing the genomes of closely related strains across host species, host tissue types or environments can be used to identify genes, including virulence factors, that may be involved in adaptation to host or environmental factors [34,36].

For *M. ovipneumoniae*, comparative genomic analyses across strains have been limited in part by the scarcity of publicly available genome sequences. However, studies using sequence data from a small subset of the genome (i.e. fewer than ten genetic loci through MLST) have identified two separate, highly divergent *M. ovipneumoniae* clades that are strongly associated with domestic sheep or domestic goat host species, hereafter referred to as the ‘sheep clade’ and ‘goat clade’ [3,11, 37]. Genetic diversity is high both between and within these two clades [3,4, 11, 37, 38], and disease outcomes vary widely across populations and host species for both clades [19,22]. Several studies have found evidence that sheep clade strains cause more severe pneumonia in bighorn sheep than goat clade strains [39,42]. In contrast, a sheep clade strain detected across multiple populations of barren-ground caribou (*Rangifer tarandus granti*) and Dall’s sheep in Alaska has not been associated with population-wide mortality events [43]. This variation in disease outcomes could be driven by strain-specific differences in pathogenicity mechanisms that evolved due to adaptive differences across host species, climates or other environmental variables. Alternatively, variation in disease outcomes could be driven by other factors such as host resistance, coinfections or environmental variation.

To gain insight into the genomic basis of pathogenicity in *M. ovipneumoniae*, we generated and compared whole-genome sequences of this bacterium across multiple geographic regions, host species and clades. We used several approaches to identify genes that may be involved in adaptation to within-host conditions or habitats, which could be responsible for differences in pathogenicity across strains and host species. First, we identified genes showing evidence for homologous recombination, positive selection or diversifying selection, each of which could be indicative of adaptive evolution (e.g. [44,47]). We also identified genes for which presence or absence was significantly associated with host species, which could indicate host adaptation. The identification of these genes provides insight into potential mechanisms of pathogenicity for *M. ovipneumoniae*, including mechanisms that may underly differences in disease outcomes within and between host species. Finally, to evaluate whether MLST markers can be used as indicators of genome-wide variation for *M. ovipneumoniae*, we compared divergence estimates and phylogenetic relationships generated using whole-genome sequences versus those generated using sequence data from four MLST markers commonly used for genetic monitoring of this pathogen.

## Methods

### Sample collection

**Fig. 1.**
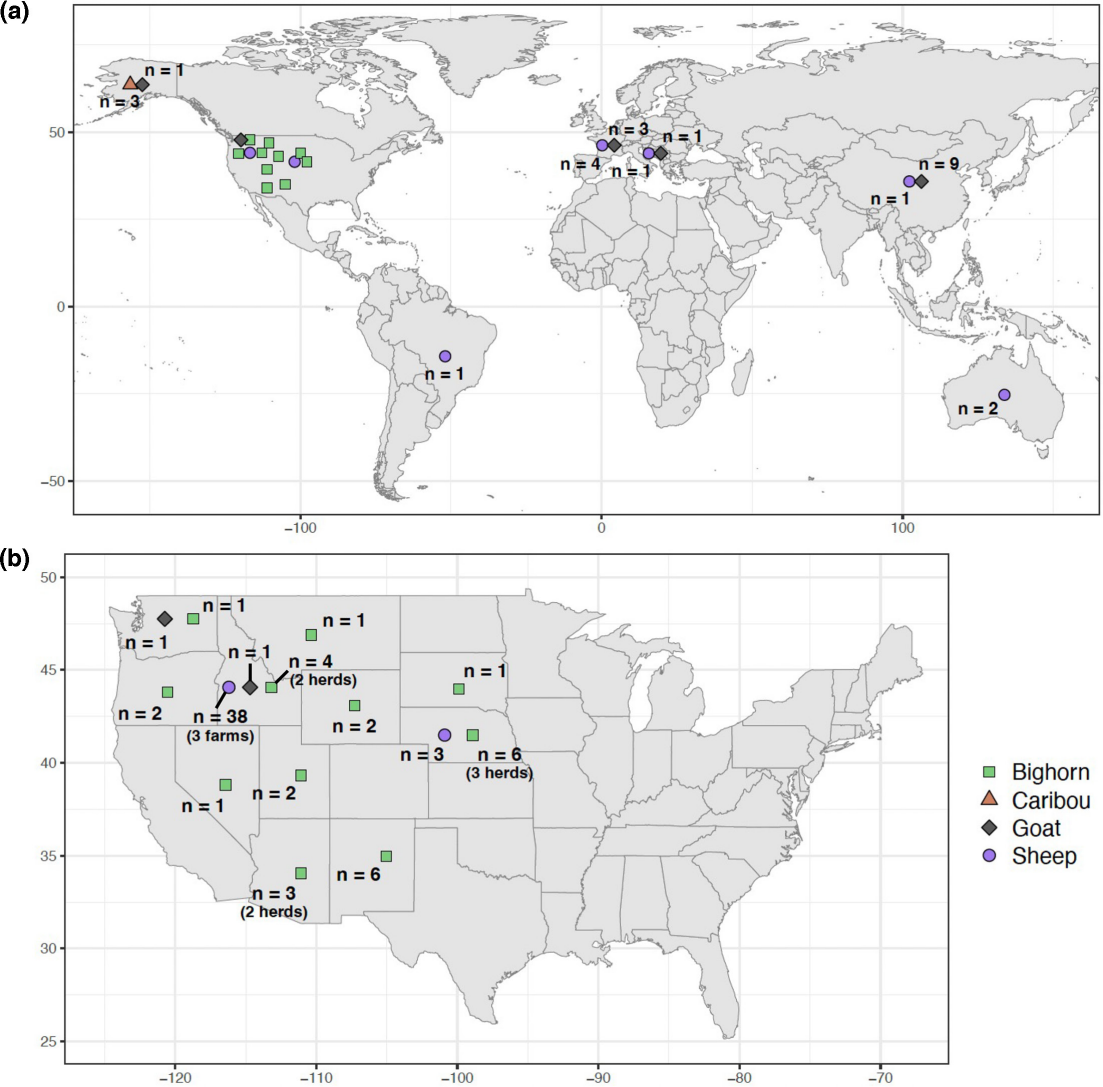
Map of sampling locations for each host species across (a) the world and (**b**) 48 US states. For US states, the number of herds or farms is shown if more than one was sampled. Locations are shown as the centre of the country or state in which the sample was collected, rather than exact sampling location coordinates. The sampling location for the captive bighorn sheep is reported as Nevada; this sheep was infected in South Dakota with a Nevada-origin bighorn sheep strain.

Deep nasal swab samples were collected from domestic sheep, domestic goats, bighorn sheep and caribou across ten US states (Fig. 1, Tables 1 and S1, available in the online version of this article). After collection, swabs were immediately transferred to cryotubes containing a preservation medium composed of either sterile PBS with 20 % v/v glycerol or a growth medium such as tryptic soy broth (TSB) with at least 15 % v/v glycerol. When necessary, these cryotubes were stored at −20 °C or −80 °C until the enrichment procedure. Samples collected in the field were shipped on ice or dry ice. Samples were collected by state wildlife management agencies or universities in accordance with animal care guidelines and protocols or were collected under University of Idaho Institutional Animal Care and Use Committee Protocol IACUC-2019-69.

**Table 1.** Total sample sizes of *M. ovipneumoniae* for each host species and sampling location. ‘SDSU (Captive)’ = captive bighorn sheep that was infected at South Dakota State University with a Nevada-origin bighorn sheep strain. Unk. = unknown

Host species	Country or US state	Site	*N*
Domestic sheep	Australia	Queensland	2
	Bosnia and Herzegovina	Hercegovacko-neretvanski	1
	Brazil	Rio de Janeiro	1
	China	Hohhot	1
	France	Unk.	3
		Vaucluse	1
	USA: Idaho	Farm 1	14
		Farm 2	11
		Farm 3	13
	USA: Nebraska	Farm 4	3
Bighorn sheep			
	USA: Arizona	Bighorns	1
		Vermillions	2
	USA: Idaho	Lost River	3
		South Beaverhead	1
	USA: Montana	Elkhorn Mountains	1
	USA: Nebraska	Barrell Butte	2
		Fort Robinson	2
		Hubbard’s Gap	2
	USA: New Mexico	Taos Pueblo	6
	USA: Nevada	SDSU (Captive)	1
	USA: Oregon	Lostine	2
	USA: South Dakota	Rapid Creek	1
	USA: Utah	Antelope Island	2
	USA: Washington	Black Butte, Hells Canyon	1
	USA: Wyoming	Whiskey Mountain	2
Caribou	USA: Alaska	Nelchina	3
Domestic goat	China	Gansu	1
		Sichuan	8
	France	Unk.	3
	Bosnia and Herzegovina	Hercegovacko-neretvanski	1
	USA: Alaska	Farm 5	1
	USA: Idaho	Farm 6	1
	USA: Washington	Farm 7	1
		Farm 8	1
Total			99

We also downloaded genome assemblies or raw Illumina shotgun sequence data from NCBI for 25 strains collected from domestic sheep and goats and bighorn sheep from 6 countries (Australia, Bosnia and Herzegovina, Brazil, China, France and USA; Table S1). Of these assemblies, 19 were sequenced using Illumina technology, 5 using Pacific Biosciences (PacBio) technology and 1 using Roche 454 technology (Table S1). These included two assemblies from the type strains of *M. ovipneumoniae* obtained from the American Type Culture Collection (ATCC) [product number 29149, strain NCTC 10151 (Y98)]. These strains were originally isolated from domestic sheep in Australia in 1971 [48,49] (Table S1). For strains that only had raw Illumina shotgun data on NCBI (*n*=6 from France [3]; Table S1), we assembled the genomes using the method described later for Illumina data. One additional assembly from a domestic sheep in New Zealand was also downloaded from NCBI (GenBank GCA_009756935.1), but this assembly was not included in the final analyses due to poor quality (i.e. low completeness and high contamination based on assessment methods described further later).

### Sample culture and enrichment

To selectively enrich *M. ovipneumoniae*, inoculated nasal swabs were subjected to a two-step procedure. First, swabs were inoculated in 4 ml mycoplasma broth (product #R102, Hardy Diagnostics, CA, USA) and incubated at 37 °C in a shaking incubator (200 r.p.m.) for 4 days. Then, 100 µl aliquots of these enrichment cultures were each transferred into 2 ml of SP4 broth with glucose (product #U86, Hardy Diagnostics, CA, USA) and re-incubated under the same conditions either until the pH indicator in the culture medium turned orange/yellow (indicating potential growth of glucose-fermenting *Mycoplasma*) or until 15 days had elapsed with no colour change observed. Turbid broth cultures indicating the growth of contaminant bacteria, as well as cultures that never turned colour, were discarded. After SP4 broth incubation, 1 ml aliquots were removed and centrifuged (20 min at 14 000 g), and the resulting cell pellets were stored at −20 °C. For the two samples collected from Wyoming in 2021, a separate protocol was used for culturing *M. ovipneumoniae*, as described elsewhere [50].

### DNA extraction and sequencing

Total genomic DNA was extracted from the frozen cell pellets using the QIAamp DNA Mini Kit (Qiagen). Confirmation of the presence of *M. ovipneumoniae* was obtained by quantitative PCR (qPCR) targeting the 16S rRNA gene, using the primers 226 F_new and LMR1, and the 253 P probe, using protocols previously described [6].

Whole-genome shotgun sequencing was performed for all samples that had a qPCR Ct value below 30 (when the threshold was manually set at 0.02). Whole-genome shotgun libraries were prepared using Illumina Nextera DNA kits and sequenced on an Illumina MiSeq using the v3 600 cycle sequencing kit. Oxford Nanopore Technologies (ONT) sequencing was also performed for samples with sufficient genomic DNA remaining after Illumina sequencing, and for which analysis of Illumina sequencing data indicated relatively low contamination levels (i.e. the majority of sequence reads were classified as *Mycoplasma*; this analysis is described later). Barcoded ONT libraries were created using the SQK-LSK109 library prep kit, and sequencing was performed for 48 h on FLO-MIN106D flowcells, with 12 samples per flowcell. All sequencing was performed at the University of Idaho Institute for Interdisciplinary Data Sciences Genomics and Bioinformatics Resources Core.

### Genome assembly

Raw Illumina sequence reads were cleaned using HTStream (https://ibest.github.io/HTStream) to filter PhiX reads, PCR duplicates, adapter sequences and low-quality reads. Because *M. ovipneumoniae* is a fastidious micro-organism known to present challenges for enrichment culture from nasal swabs, we tested for potential contamination with non-*Mycoplasma* sequences using the metagenomic classifier Centrifuge v.1.0.4 (51) to classify the cleaned Illumina sequence reads. For subsequent analyses, we only retained samples for which >95 % of bacterial sequence reads were classified as *Mycoplasma*.

For samples with ONT sequence data, raw ONT sequence reads were demultiplexed by barcode and base-called using Guppy v.4.2.2 (Oxford Nanopore Technologies Ltd.). Assembly was performed using Trycycler v.0.4.1 [52], which incorporates methods for identifying and removing potential contamination from assemblies by removing assembled contigs that are not the expected read depth and length (approximately 1 MB for *M. ovipneumoniae*; Supplementary Text). Assemblies were polished using cleaned Illumina sequence reads with Pilon v.1.23 [53] (Supplementary Text). For samples with only Illumina sequence data and no ONT sequence data, cleaned Illumina reads were assembled *de novo* using SPAdes v.3.13.1 [54] using the parameters ‘--careful’ and ‘-k 33,77,127’ (Supplementary Text).

All assemblies, including those from prior studies, were analysed using the lineage-specific workflow in CheckM v.1.0.13 [55] to evaluate contamination and completeness. This programme determines whether expected lineage-specific genes are present in the assemblies. For subsequent analyses, we only retained genome assemblies with low contamination (<5 %) and high completeness (>98 %).

### Pangenome characterization

Genome-wide divergence between each pair of samples was estimated by calculating average nucleotide identity (ANI) for all orthologous genes shared between each pair of genomes using FastANI v.1.32 [56]. A threshold ANI value of 95 % is often used as a cutoff for the distinction of separate species [56]. All assemblies, including those from prior studies, were initially annotated using Prokka v.1.14.5 [57] with the ‘-gcode’ parameter set to 4 to enable appropriate translation for *Mycoplasma* species. This programme uses Prodigal v.2.6.3 [58] for gene prediction and then predicts gene products by comparing gene sequences against reference databases using BLAST+ v.2.9.0 [59] and HMMER v.3.2.1 [60]. We did not perform analyses to identify plasmids because our assembly method for ONT sequences included a step to remove contigs that were not the expected chromosome length (approximately 1 MB; described earlier). This step provided an effective way to remove potential contaminating sequences but also would have removed any plasmids that were present in the samples. This step was used because we sequenced enrichment cultures rather than purified bacterial isolates, which increased the probability of contaminant DNA sequences. Notably, plasmids have not been reported in *M. ovipneumoniae* assemblies published thus far [5].

We next determined the pangenome (i.e. the full set of genes in the dataset) and determined which genes were present or absent in each sample using Roary v.3.13.0 [61]. For this analysis, we used the PRANK [62] codon-aware alignment option and a minimum per cent identity of 92 %, rather than the default value of 95 %, to account for the relatively high level of pairwise genetic variation across samples identified by the ANI analysis (described further in the Results section). We also used the ‘-z’ option to generate sequence alignments for each gene. We then used the sidekick.py script from the HERO pipeline (Park C, Andam C. HERO Github https://github.com/therealcooperpark/hero) to replace the gene-specific headers in the Roary gene alignments with the sample names.

We also annotated the genomes using EggNOG-mapper v.2.1.9 [63,64]. This approach can outperform Prokka in the number of genes assigned a gene product, especially for non-model organisms including *M. ovipneumoniae*, due to the use of a more comprehensive database [65,66]. For this analysis, we used the Diamond search strategy [67] and the associated Diamond reference database provided by EggNOG-mapper. As input for this analysis, we used the Roary output file pan_genome_reference.fa, which includes representative sequences from each gene identified by Prodigal during the gene prediction step of the Prokka analysis.

We also used the Roary output to determine the core and accessory genes for our dataset. Core genes are defined as those present in 99–100 % of strains, and soft core genes are those present in 95–99% of strains. Accessory genes are comprised of shell genes and cloud genes, where shell genes are those present in 15–95 % of strains and cloud genes are those present in <15 % of strains. We also evaluated the total number of core and accessory genes as a function of sample size using Roary. These analyses allow assessment of whether the sample size in our study is sufficient to characterize the core genome and the pangenome of the species. We initially performed Roary analyses using all samples and then separately for the samples belonging to each of two highly divergent clades identified by our phylogenetic analyses (‘sheep clade’ and ‘goat clade,’ described later).

### Virulence genes from prior studies

To identify potential virulence genes in our assemblies, we first used VFanalyzer [68] to identify genes that have been associated with virulence in prior studies for *Mycoplasma*. This programme compares genetic sequences against the virulence factor database (VFDB, http://www.mgc.ac.cn/VFs/) [69]. As input for this analysis, we used the Roary output file pan_genome_reference.fa, after converting nucleotide sequences to aa sequences. As described earlier, this file includes representative sequences from each gene identified by Prodigal. We also used the lists of genes identified for each assembly by Prokka and EggNOG-mapper (described earlier) to determine whether any additional genes were present that were not identified by VFanalyzer but that have been implicated as potentially involved in virulence for *M. ovipneumoniae* and other *Mycoplasma* species in prior studies [23,66, 70].

### Phylogenetic analysis

We conducted phylogenetic analyses using a nucleotide alignment of all core genes identified using PRANK [62] in Roary. We generated a maximum likelihood tree with RAxML-NG v.1.0.0 [71] with the GTR+Gamma model by first identifying the best-scoring tree from 50 maximum likelihood trees generated from 25 random and 25 parsimony-based starting trees and then performing bootstrapping for the best tree using default settings. The assemblies used in this study varied widely in the numbers of contigs, with most assemblies that were generated using long-read technologies (PacBio, ONT or Roche 454) being circularized and all assemblies that were generated using short-read technology (Illumina) having multiple contigs (Table S1). Therefore, we also evaluated the potential influence of sequencing technology on phylogenetic analyses by performing separate analyses using only short-read or long-read assemblies. For these analyses, core genome identification and phylogenetic analyses were performed using the same methods as described earlier for the full dataset.

### Adaptive variation

We used several approaches to identify genes potentially involved in adaptation. First, we identified genes with high levels of homologous recombination, which could be indicative of adaptive evolution [72,74]. We used fastGEAR [75] to quantify recombination for each core and accessory gene, using the gene sequence alignments generated by Roary. FastGEAR first characterizes population structure by identifying lineages using Bayesian analysis of population structure (BAPS) [76] followed by a hidden Markov model approach. Next, fastGEAR identifies recent recombinations (occurring at the tips of the phylogeny) and ancestral recombinations (affecting entire lineages) on a gene-by-gene basis. For this approach, recombination sources could be lineages within the dataset, or unknown lineages not represented in the dataset.

Another approach we used to identify genes potentially involved in adaptation was to investigate core genes experiencing positive or diversifying selection. For this approach, we estimated the ratio of nonsynonymous to synonymous substitutions (*dN*/*dS*) for each pair of genomes for each core gene using the codeml function in PAML v.4.9c [77], with alignments for each core gene extracted from the core genome alignment generated by Roary. Values of *dN*/*dS* >1 indicate positive or diversifying selection, whereas values of *dN*/*dS* <1 indicate purifying selection. Genes under positive or diversifying selection are candidates for involvement in adaptive differences across strains. For this analysis, we set the codeml ‘icode’ parameter to 3 to enable appropriate translation for *Mycoplasma* species. After generating pairwise *dN*/*dS* values, we filtered out all pairwise comparisons with *dN*/*dS*=99 (which designates a value that could not be calculated accurately due to a *dS* value near 0 [78]) and all pairwise comparisons with *dN*=0 and *dS* <0.01 (to avoid comparisons between genome sequences from the same population [79]). In addition, we filtered out all pairwise comparisons for genes with poor nucleotide alignments, which we defined as alignments with <70 % of nucleotides remaining after gaps (i.e. alignment sites with no nucleotides for one or more samples) were removed by codeml.

The next approach we used for identifying genes potentially involved in adaptation was a ‘pangenome-wide association study’ (pan-GWAS) approach implemented in Scoary [80] to identify genes for which presence or absence is significantly associated with one of the hosts (domestic sheep, domestic goats, bighorn sheep and caribou) or each of the two major clades that were identified in our phylogenetic analysis (i.e. the ‘sheep clade’ and the ‘goat clade’; described later). The approach used by Scoary accounts for population stratification and can have strong statistical power even with relatively small sample sizes [80,81]. For this analysis, we focused on genes that had been assigned a gene product by EggNOG-mapper or VFanalyzer. We defined a gene as ‘present’ in a genome if at least one gene had been assigned to that gene product in the genome. Because Scoary requires binary traits, we conducted three tests comparing genomes from the following pairs of groups: sheep versus goat clades (caribou samples were excluded from this analysis since these assemblies had significantly fewer genes than all other sheep clade assemblies, as described later), domestic sheep versus bighorn sheep hosts within the sheep clade, and caribou samples versus the rest of the sheep clade. We used a Bonferroni correction value of 0.05 as the significance threshold.

Because two of the genes identified as host-associated by the Scoary analysis were part of the clustered regularly interspaced short palindromic repeats and associated proteins (CRISPR-Cas) system (described further in the Results section), we also investigated whether each genome assembly contained a functional CRISPR-Cas system by identifying high-confidence CRISPR regions (evidence level=4) using CRISPRCasFinder v.4.3.2 [82,84]. The presence of a functional CRISPR-Cas system in *Mycoplasma* is expected to be characterized by the presence of CRISPR arrays and the genes *cas1*, *cas2* and *cas9* [85].

To determine whether the genes identified as potential targets of selection were enriched for certain gene functions, we used Gene Ontology (GO) enrichment analyses implemented in the R package topGO [86]. These analyses were performed using the list of annotated genes in the pangenome as a reference, including all genes that had been assigned a gene product by EggNOG-mapper. We assigned GO terms to each gene in the pangenome using the UniProtKB database [87], including GO terms related to biological processes, molecular functions and cellular components. We accepted GO terms that had been assigned to bacteria and that had been manually annotated (Swiss-Prot records). For genes that had no Swiss-Prot records, we used GO terms that had been assigned to bacteria in the computationally annotated TrEMBL database. Genes for which putative gene products had not been identified by EggNOG-mapper were removed from gene lists prior to these analyses. Significant enrichment of GO terms was determined using Fisher’s exact test with a significance threshold of *P*<0.05.

### Comparing pathogen monitoring techniques: MLST versus whole genomes

To compare between-sample pairwise genetic divergence values calculated using MLST sequences versus those calculated using genome assemblies, we first used BLAST+ v.0.2.9.0 [88] to extract sequences from each assembly for each of the four MLST markers commonly used for *M. ovipneumoniae* surveillance. These markers include partial sequences from the 16S–23S IGS, the small ribosomal subunit (16S rRNA) and genes encoding rpoB and gyrB [11]. We conducted this analysis using a custom reference database we created using representative *M. ovipneumoniae* sequences obtained from a prior study for each MLST marker [11]. We then aligned the extracted sequences for each MLST marker using the Geneious Alignment algorithm in Geneious v.11.1.5 (Auckland, New Zealand). For the IGS marker, a portion of the alignment was removed due to the presence of a large number of indels, which led to poor alignment quality that could cause inaccurate estimates of pairwise divergence. We then used the ‘dist.gene’ function in the R package ape [89] to calculate ANI between each pair of sequences for each marker, for the four markers concatenated and for all markers except IGS concatenated (since removal of the IGS indel region could have influenced the results). We then plotted the ANI values calculated using MLST markers versus those calculated using the genome assemblies as described earlier. We also generated maximum likelihood phylogenies with concatenated MLSTs using RAxML-NG. For this analysis, we partitioned the sequence data by locus and used the GTR+Gamma model for each partition and used the same tree searching strategy and bootstrapping methods as described earlier for the genome assemblies.

## Results

### Sample sizes

After quality filtering of genome assemblies, we retained a total of 99 *M*. *ovipneumoniae* genome assemblies, including 74 that we sequenced and assembled for this study (Supplementary Text) and 25 that we obtained from NCBI (Fig. 1, Tables 1 and S1). Of these, 50 assemblies originated from samples collected from domestic sheep from 2 US states and 5 additional countries (Australia, Bosnia and Herzegovina, Brazil, China and France), including two assemblies from the *M. ovipneumoniae* type strains that were collected from domestic sheep in Australia. In addition, 17 assemblies originated from domestic goats from 3 US states and 3 additional countries (Bosnia and Herzegovina, China and France), 28 from wild bighorn sheep from 9 US states, 1 from a bighorn sheep infected in captivity with a Nevada-origin bighorn sheep strain and 3 from caribou from Alaska (Tables 1 and S1). The *M. ovipneumoniae* type strains had been collected in 1971 and one assembly originated from a sample collected from a domestic goat in China in 1998; all other samples had been collected between 2007 and 2021 (Table S1).

### Pangenome characteristics: full dataset

**Fig. 2.**
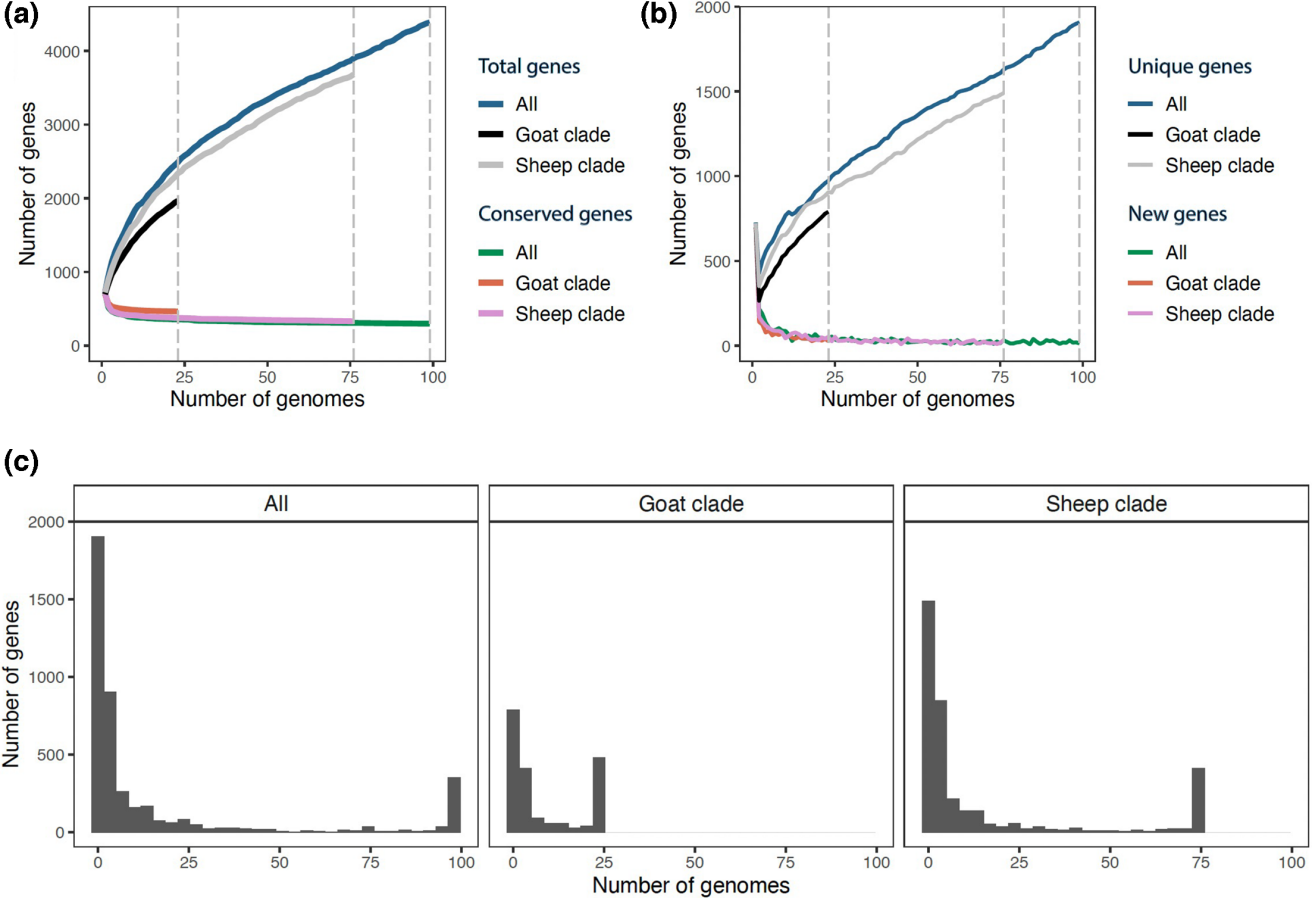
Pangenome characteristics of *M. ovipneumoniae* assemblies. Results are shown for all assemblies (*n*=99), only the assemblies in the sheep clade (*n*=76) and only the assemblies in the goat clade (*n*=23). (**a**) The numbers of genes in the core genome (‘conserved genes’) and the pangenome (‘total genes’) as a function of sample size. (**b**) The numbers of ‘new genes’ (genes not found in previously sampled assemblies) and ‘unique genes’ (genes found in only one of the sampled assemblies) as a function of sample size. (**c**) The number of genes shared by different numbers of genomes.

The *M. ovipneumoniae* pangenome (i.e. the total number of predicted genes across all assemblies) identified by Roary analysis totalled 4387 (Table S2). The number of core genes was 298 and the number of soft core genes was 79; together, the core and soft core genes represent only 8.6 % of the pangenome. For the accessory genes, the number of shell genes was 643 and the number of cloud genes was 3367. The evaluation of pangenome content as a function of sample size showed that the number of conserved genes levelled off around ten genomes, indicating that the sample size in our study was sufficient to characterize the core genome (Fig. 2). In contrast, the number of accessory genes continued to increase as more genomes were added, indicating that our dataset does not characterize all the accessory genes for *M. ovipneumoniae* (Fig. 2). The high number of accessory genes relative to core genes could indicate strong ability of this pathogen to adapt to within-host conditions, as there is growing evidence that accessory genes facilitate adaptation [90,91].

Only 726 of the 4387 predicted genes in the pangenome (16.5 %) were assigned a putative gene product by Prokka, with the remaining genes being assigned the category of ‘hypothetical protein’ (Table S2). In contrast, a much higher percentage of the genes in the core genome were assigned a putative gene product (217 of the 298 core genes, 72.8 %). A total of 138 annotated genes were present more than once in the pangenome, with the number of copies ranging from 2 to 55 (mean=3.43) (Table S2). The presence of genes with multiple copies in the pangenome indicates the presence of either paralogous genes or highly divergent copies of orthologous genes that were split into separate genes by the Roary analysis. When using EggNOG-mapper to annotate the genes, a higher percentage of the predicted pangenome genes were assigned a putative gene product (938 genes, 21.4 %) than when using Prokka, including 243 (81.5 %) of the core genes (Table S2). A total of 179 genes were present more than once, with the number of copies present ranging from 2 to 47 (mean=3.77) (Table S2). Since EggNOG-mapper assigned products to more genes than Prokka, we used EggNOG-mapper annotations for subsequent analyses investigating adaptive genomic variation (described later).

### Potential virulence genes

VFanalyzer identified ten genes that are potential *Mycoplasma* virulence factors in our genome assemblies, including enolase (*eno*), hemolysin (*hlyA*), nuclease (*nuc*) and seven genes involved in adhesion (*lppT*, *p102*, *p216*, *p97*, *pdhB*, *plr/gapA* and *tuf*) (Tables 2 and S2). For 5 of these genes, 1 copy was found in all 99 genome assemblies (*eno*, *plr/gapA*, *hlyA*, *pdhB* and *tuf*), and for 3 genes, at least 1 copy was found in 92–97 assemblies (*lppT*, *p102* and *nuc*) (Table S2). For the remaining 2 genes, at least 1 copy was found in 74 assemblies (*p97*) or 10 assemblies (*p216*) (Table S2). Gene annotation with Prokka and EggNOG-mapper also identified additional genes that have been proposed as virulence factors in previous studies of *M. ovipneumoniae* and other *Mycoplasma* species (Tables 2 and S2). These included genes involved in glycerol catabolism and the associated production of hydrogen peroxide (*glpF*, *glpK*, *glpO*, *glpQ*, *gtsA*, *gtsB* and *gtsC*), hemolysis (*acpD*), adhesion (*mgpA*) and inflammatory responses (*atpF*) [23,66, 70]. Each of these additional genes was present in all 99 of our genome assemblies, with the exception of *glpQ*, which was present in only 34 assemblies.

**Table 2.** Candidate virulence genes identified in 99 *M. ovipneumoniae* genomes obtained from sheep, goats and caribou. Potential mechanism = proposed virulence mechanism for the gene. Analysis* = in silico analysis method that identified the putative virulence gene. Sheep clade, goat clade and caribou subclade = percent of assemblies within the clade for which the gene was present (sheep clade excludes the caribou subclade assemblies)

Gene	Potential mechanism	Analysis	Goat clade	Sheep clade	Caribou subclade
*eno*	Enzyme	VFDB	100	100	100
*hlyA*	Hemolysin	VFDB	100	100	100
*nuc*	Nuclease	VFDB	95.7	98.6	100
*lppT*	Adhesion	VFDB, Scoary (caribou vs. sheep)	87.0	98.6	0
*p102*	Adhesion	VFDB	87.0	100	100
*p216*	Adhesion	VFDB	13.0	5.5	100
*p97*	Adhesion	VFDB	47.8	82.2	100
*pdhB*	Adhesion	VFDB	100	100	100
*plr/gapA*	Adhesion	VFDB	100	100	100
*tuf*	Adhesion	VFDB	100	100	100
*glpF*	Glycerol metabolism	Literature	100	100	100
*glpK*	Glycerol metabolism	Literature	100	100	100
*glpO*	Glycerol metabolism	Literature	100	100	100
*glpQ*	Glycerol metabolism	Literature, Scoary (goat vs. sheep)	100	21.9	0
*gtsA*	Glycerol metabolism	Literature	100	100	100
*gtsB*	Glycerol metabolism	Literature	100	100	100
*gtsC*	Glycerol metabolism	Literature	100	100	100
*acpD*	Hemolysis	Literature	100	100	100
*mgpA*	Adhesion	Literature	100	100	100
*atpF*	Inflammatory response	Literature	100	100	100
*rnhB*	H_2_O_2_ production	Scoary (goat vs. sheep)	39.1	0	0
*baiH*		Scoary (caribou vs. sheep)	100	100	0
*gcdH*		Scoary (caribou vs. sheep)	100	100	0
*lemA*		Scoary (caribou vs. sheep)	100	100	0
*lplA-2*		Scoary (caribou vs. sheep)	100	100	0
*nagA*	Carbon metabolism	Scoary (caribou vs. sheep)	100	100	0
*nagC*	Carbon metabolism	Scoary (caribou vs. sheep)	100	100	0
*nagE*	Carbon metabolism	Scoary (caribou vs. sheep)	100	100	0
*nanA*	Carbon metabolism	Scoary (caribou vs. sheep)	100	100	0
*nanE*	Carbon metabolism	Scoary (caribou vs. sheep)	100	100	0
*pgmB*		Scoary (caribou vs. sheep)	100	100	0
*ruvB*		Scoary (caribou vs. sheep)	100	100	0
*smf*		Scoary (caribou vs. sheep)	100	100	0
*tdh*		Scoary (caribou vs. sheep)	100	100	0
*treP*	Carbon metabolism	Scoary (caribou vs. sheep)	100	100	0
*ulaA*	Carbon metabolism	Scoary (caribou vs. sheep)	100	100	0
*cas1*	CRISPR-Cas	Scoary (caribou vs. sheep)	100	97.3	0
*cas2*	CRISPR-Cas	Scoary (caribou vs. sheep)	100	97.3	0
*fruA*	Carbon metabolism	Scoary (caribou vs. sheep)	100	97.3	0
*malC*	Carbon metabolism	Scoary (caribou vs. sheep)	100	97.3	0
*hsdR*		Scoary (caribou vs. sheep)	91.3	95.9	0
*malK*	Carbon metabolism	Scoary (caribou vs. sheep)	100	95.9	0
*pr2*		Scoary (caribou vs. sheep)	100	95.9	0

1*“‘VFDB’ = gene identified by comparing genome sequences against the virulence factor database (VFDB);. ‘Literature’ = gene has been proposed as a virulence factor for *M. ovipneumoniae* in previous studies;. ‘Scoary’ = gene for which presence/absence was significantly associated with one of the host species through a comparison of the two host species listed in parentheses.

### Phylogenetic analysis

**Fig. 3.**
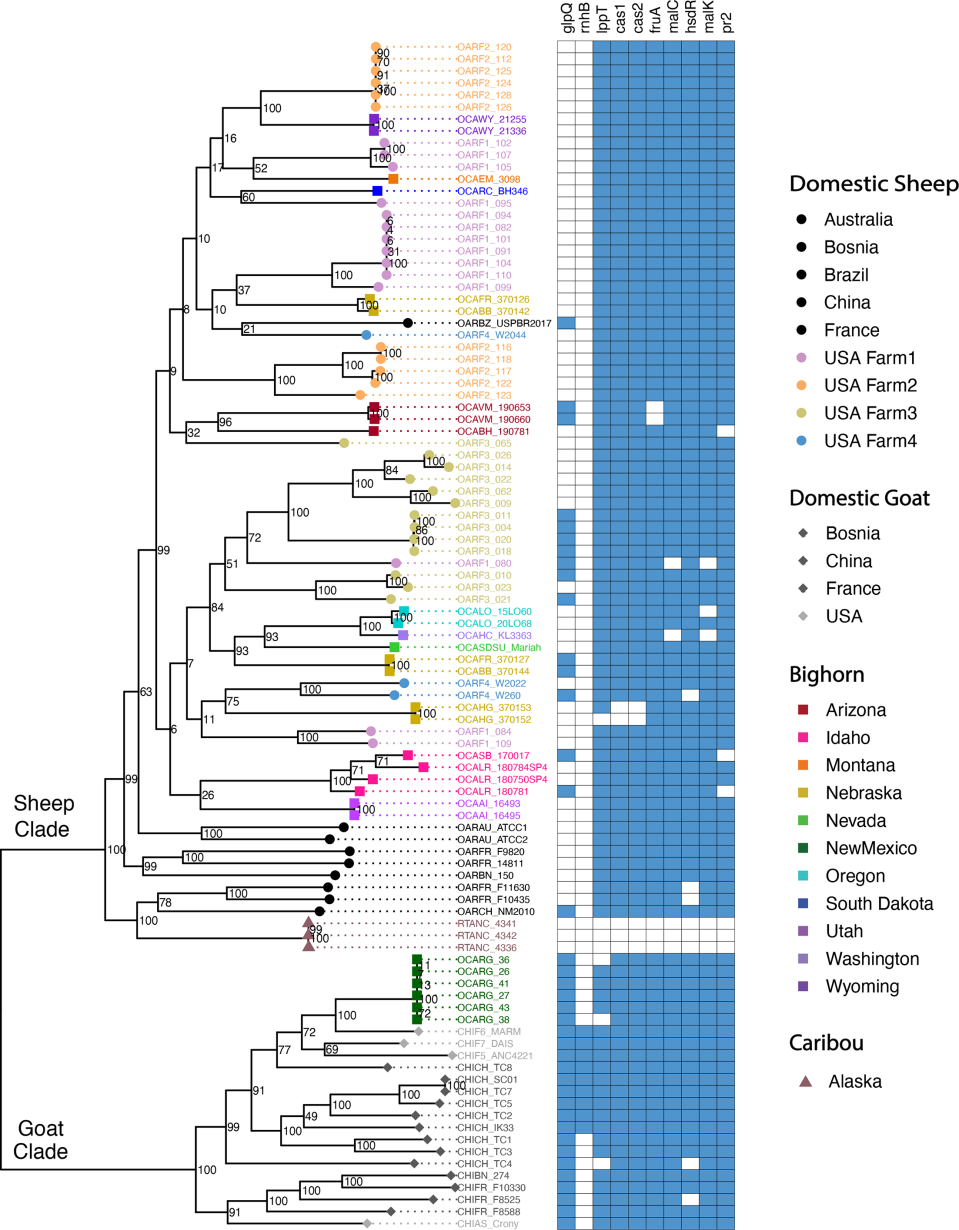
Maximum likelihood phylogeny of core genome sequences for 99 *M. ovipneumoniae* assemblies and presence (blue) or absence (white) in each assembly for genes that were significantly associated with host species based on Scoary analysis (Table 1). Significantly associated genes that were found in 0 % of caribou subclade assemblies and 100 % of all other assemblies are not shown (Table 1). An interactive phylogeny is available at https://nextstrain.org/community/narratives/kimandrews/Movi.

Phylogenetic analyses of the core genes for the full dataset (both long- and short-read assemblies) indicated high genetic diversity across *M. ovipneumoniae* and the presence of two highly divergent clades, which were strongly associated with host species. All assemblies from domestic sheep fell in one clade (‘sheep clade’), and all assemblies from domestic goats fell in the other clade (‘goat clade’) (Fig. 3). All assemblies from bighorn sheep and caribou fell in the sheep clade, with the exception of six assemblies from a single free-ranging bighorn sheep population in New Mexico, which were in the goat clade.

Within the sheep clade, the core genome tree placed all domestic and wild sheep assemblies from the USA and Brazil within a subclade with high bootstrap support (value=99) (Fig. 3). This subclade had minimal phylogenetic clustering associated with geographic distance between herds; for example, each farm had assemblies that clustered in separate, divergent phylogenetic lineages, and some bighorn sheep herds in the same state had highly divergent assemblies, despite their close geographic proximity (e.g. herds OCABB and OCAFR in Nebraska). In addition, US/Brazilian sheep subclade assemblies did not cluster separately by domestic or wild sheep hosts.

The core genome tree indicated the three caribou assemblies were genetically similar to each other and placed these assemblies within the sheep clade, in a separate subclade from the rest of the US samples, together with two domestic sheep assemblies from France and one from China (Fig. 3). However, the three caribou assemblies were highly divergent from the other assemblies in the subclade, indicating that no close relatives to the caribou assemblies are present in our dataset.

Core genome phylogenies generated using only assemblies from short-read (Fig. S1) or long-read (Fig. S2) sequencing technologies were consistent with the phylogeny from the full dataset (Fig. 3). These results indicate that differences in sequencing technology across genome assemblies did not influence the phylogenetic results.

### Genome-wide similarity across assemblies

**Fig. 4.**
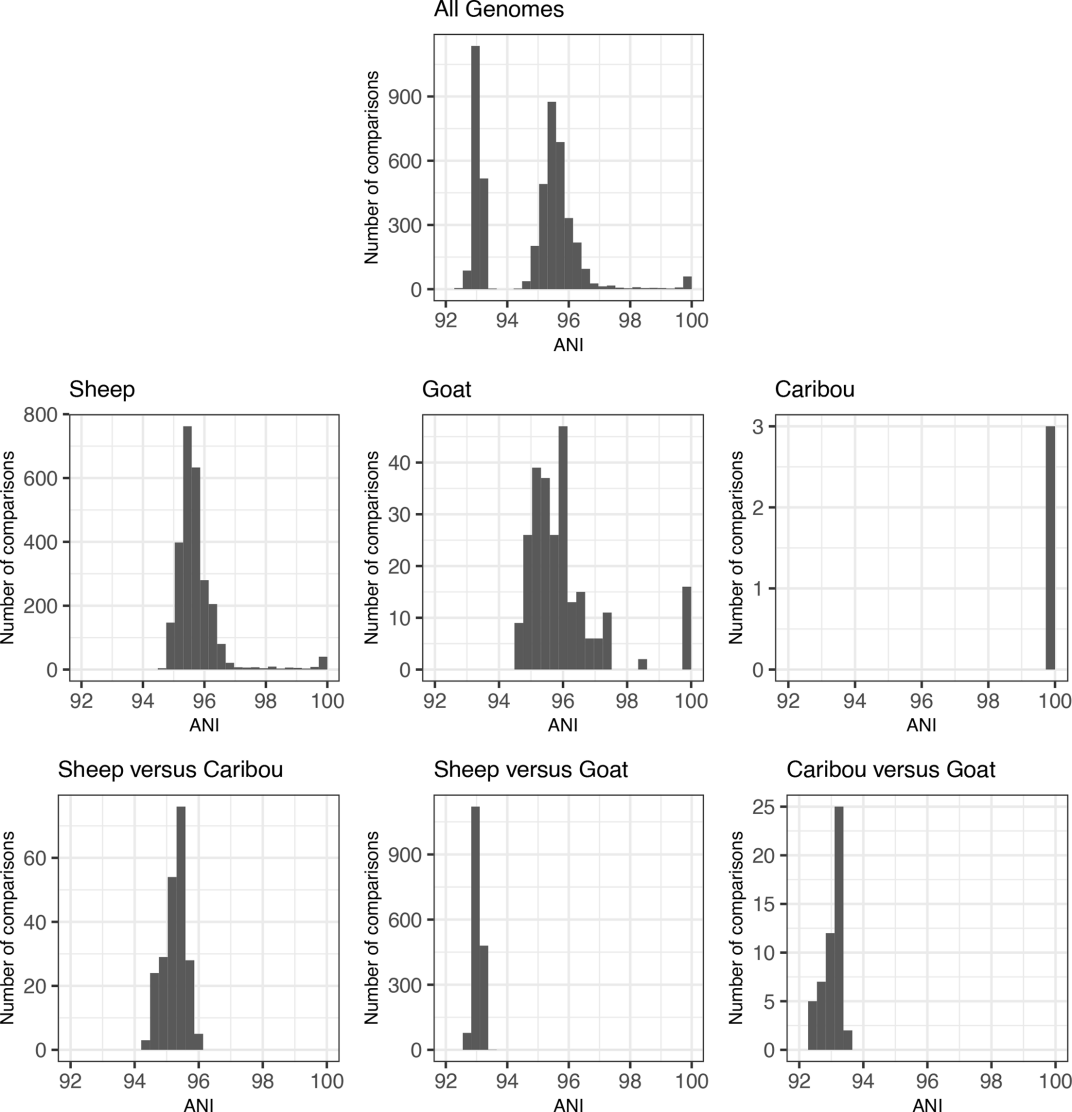
ANI for all orthologous genes shared between each pair of 99 *M. ovipneumoniae* genomes, including pairwise comparisons across all genomes (‘All Genomes’) and pairwise comparisons between and within three groups of samples: ‘sheep’ clade (including all domestic and bighorn sheep assemblies that were assigned to this clade based on phylogenetic analysis results, but excluding caribou assemblies for comparative purposes); ‘goat’ clade (including all assemblies assigned to this clade, i.e. all domestic goat assemblies and some bighorn sheep); and ‘caribou’ subclade (including all assemblies collected from caribou; these formed a subclade within the sheep clade). The greatest genome-wide differences are between the sheep and goat clades; all pairwise comparisons within these clades have ANI >94 %, and all pairwise comparisons between these two clades have ANI <94 %.

Estimates of genome-wide similarity across all assemblies indicated the presence of two genetically divergent groups within the full dataset, with ANI values showing a bimodal distribution comprised of one peak around 93 % and another around 95.5 % (Fig. 4). Within the clades identified by phylogenetic analysis (sheep clade, goat clade and caribou subclade), most ANI values were >94 % across pairwise comparisons (Fig. 4). However, all ANI values were <94 % for pairwise comparisons between the goat clade and the other groups. These results were consistent whether using the full dataset (Fig. 4) or only using assemblies generated using short-read (Fig. S3) or long-read (Fig. S4) sequencing technologies. These results indicate that the bimodal distribution of ANI values was driven by the strong genetic divergence between the sheep and goat clades. Because a 95 % threshold is often used to define separate bacterial species, these results also indicate that the sheep and goat clades may represent two separate species.

### Pangenome characteristics: differences between clades and host species

Roary analysis with just the goat clade found 464 core genes, 18 soft core genes, 398 shell genes, 1087 cloud genes and 1967 total genes (Table S3). Roary analysis with just the sheep clade found 331 core genes, 85 soft core genes, 567 shell genes, 2698 cloud genes and 3681 total genes (Table S4). Rarefaction analyses indicate that the goat clade may have slightly more core genes and fewer accessory genes than the sheep clade (Fig. 2).

**Fig. 5.**
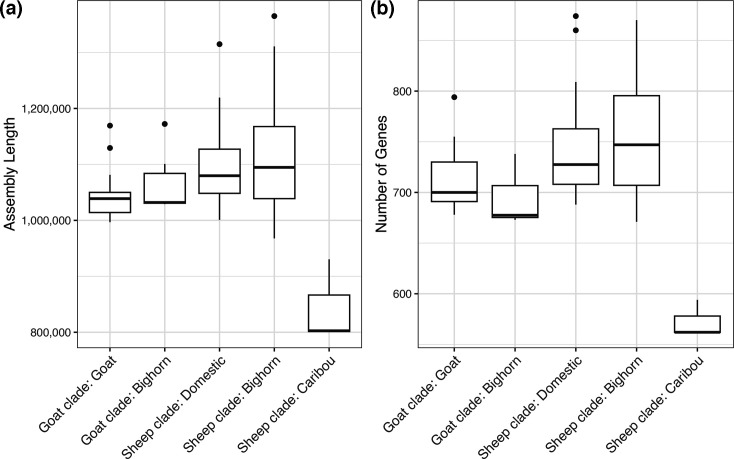
Distributions of (a) assembly lengths and (b) total numbers of genes for *M. ovipneumoniae* genome assemblies across different combinations of clades and host species.

Comparison of assembly lengths across all combinations of clades and host species indicated that the three caribou assemblies had smaller genomes than all other samples (ranging from 799 992 bp to 930 395 bp, whereas all other assemblies ranged from 967 712 bp to 1 365 101 bp) (Fig. 5a, Table S1). In addition, the caribou assemblies had fewer genes (mean=573 genes for caribou, compared to means ranging from 693 to 754 in other clade/host combinations) (Fig. 5b). Thus, the lower number of core genes in the sheep clade compared to the goat clade may have been influenced by the presence of the caribou assemblies in the sheep clade, given the lower number of genes in caribou assemblies compared to all other assemblies. Assembly lengths and numbers of genes were similar across all other combinations of clades and host species (Fig. 5). These results were consistent regardless of sequencing technology (Fig. S5).

### Adaptive variation

#### Recombination

The fastGEAR analyses with the full dataset identified 1069 genes with recent recombinations and 466 genes with ancestral recombinations, of which 384 genes had both recent and ancestral recombinations (Table S5). Of the genes with recent recombinations, 388 were annotated, and 56 of these were present multiple times in this list, indicating paralogs or divergent orthologs in the dataset. When counting only 1 copy of each annotated gene, a total of 304 unique annotated genes had at least 1 recent recombination. Of these unique genes, 33 had at least 1 copy with >10 recent recombinations. Of the genes with ancestral recombinations, 123 were annotated, of which 20 were present multiple times in the list of annotated genes, and the removal of all except 1 copy of each annotated gene resulted in 96 unique annotated genes. Of these, only 1 annotated gene (*cas9*) had a copy with >10 recombination events; this was the most abundant *cas9* gene copy (present in 70 samples) and had 23 recent recombinations and 20 ancestral recombinations.

GO enrichment analysis for the annotated genes with at least one recent recombination identified significant GO terms primarily related to transmembrane transport of carbohydrates and other organic substances, providing evidence that recent recombination has been selectively advantageous for genes related to these functions (Table S6). For the set of annotated genes with a large number (>10) of recent recombinations, GO enrichment analysis identified significant terms related to DNA replication (Table S6). For annotated genes with at least one ancestral recombination, GO enrichment analysis identified significant terms related to the same functions as for genes with recent recombinations (transmembrane transport and DNA replication). This result indicates that homologous recombination may have been selectively advantageous for genes involved in these functions throughout much of the evolutionary history of *M. ovipneumoniae* (Table S6).

#### dN/dS

Pairwise comparisons of core genome synonymous and nonsynonymous mutations across all samples identified only 22 genes with *dN*/*dS*>1 (which would indicate positive or diversifying selection) for any pairwise comparisons (Table S7). None of these genes had a large percentage of pairwise comparisons with *dN*/*dS*>1 (maximum percentage for any gene was 1.5 % when considering all comparisons, 3.8 % when only comparing goat versus sheep clade genomes, 1.36 % when only considering comparisons between sheep clade genomes, and 5.1 % when only comparing goat clade genomes; Table S8). Thus, we did not identify any core genes with strong evidence for positive or diversifying selection across our dataset based on *dN/dS* ratios.

#### Pan-GWAS

The Scoary analyses with EggNOG-mapper-annotated genes identified two genes that were overrepresented in goat clade assemblies: *glpQ* was present in all goat clade assemblies but only 22 % of sheep clade assemblies, and *rnhB* was present in 39 % of goat clade assemblies but no sheep clade assemblies (Fig. 3, Tables 2 and S9). GlpQ is involved in glycerol catabolism and the associated production of hydrogen peroxide, which is a known virulence factor for *Mycoplasma* [70,92, 93]. RnhB is involved in DNA repair and has been shown to protect bacterial cells from oxidative damage in the presence of hydrogen peroxide [94]. Within clades, the presence or absence of these genes did not correspond with phylogenetic relatedness (i.e. within the sheep clade for *glpQ* or within the goat clade for *rnhB*) (Fig. 3). These results indicate that *glpQ* and *rnhB* may be virulence factors associated with the goat clade.

Within the sheep clade, Scoary analysis identified 23 genes that were absent from the caribou assemblies but present in most or all genomes in the rest of the sheep clade, including both domestic and bighorn sheep hosts (Tables 2 and S9). One of these genes (*lppT*) had been identified as a virulence factor by VFanalyzer (described earlier) due to its involvement in adhesion to host cells for *Mycoplasma* [70]. The remaining genes were enriched for GO terms including catabolic processes, transmembrane transport and plasma membrane (Tables S10 and S11). These included multiple genes involved in membrane transport and catabolism of carbon sources alternative to glycerol. The genes absent from the caribou assemblies also included two genes that are part of the CRISPR-Cas system: *cas1* and *cas2* (Tables S9 and S12). In addition, the caribou assemblies had only partial *cas9* gene sequences (i.e. covering <50 % of the consensus sequence from the multi-sample nucleotide alignment) and fewer CRISPR repeats than most other genomes (Supplementary Text, Tables S12 and S13). These results indicate that the caribou strains do not have a functional CRISPR-Cas system, whereas most other genomes do appear to have functional systems (Supplementary Text, Table S12). No genes were overrepresented in domestic sheep versus bighorn sheep assemblies within the sheep clade.

### Comparing pathogen monitoring techniques

BLAST+ identified one copy of each of the four MLST markers in each assembly, with the exception of two assemblies that lacked a full 16S rRNA sequence and two assemblies that had two copies of gyrB (Table S14, Supplemental Results). Pairwise ANI values calculated using MLST sequences were similar to those calculated using whole genomes, ranging from 92.9 to 100 % for within-clade comparisons (compared to 94.4–100 % for whole genomes) and 92.4–99.4 % for between-clade comparisons (compared to 92.4–93.5 % for whole genomes) (Figs 4, S6 and S7). However, ANI calculated with MLST sequences had greater variance and tended to underestimate divergence compared to ANI calculated with whole genomes, although variance decreased when MLST markers were concatenated (Figs S6 and S7). Phylogenetic analysis with concatenated MLST markers separated the sheep and goat clade with strong bootstrap support (88–94 %), but most other internal nodes had low bootstrap support (Figs S8 and S9).

## Discussion

Comparative genomic analysis of 99 *M*. *ovipneumoniae* genome assemblies from 6 countries and 4 host species found high genetic diversity and a high proportion of accessory genes compared to core genes in the pangenome, characteristics which are predicted to promote adaptation [90,91]. We also found that the two major clades that were observed in previous studies to segregate by domestic host species (sheep vs. goat) are divergent enough to be considered separate bacterial species, indicating that these clades likely evolved in separate ancestral host species. Within the sheep clade, three closely related assemblies from asymptomatic wild caribou in Alaska had significantly smaller genomes and fewer genes than all other assemblies. These genomic differences indicate a separate evolutionary trajectory for this subclade resulting from geographic isolation or adaptation to a unique host or environment. However, a larger sample size from wildlife populations in Alaska is needed to confirm these results. In contrast, bighorn sheep strains did not cluster separately from domestic sheep strains in genome length comparisons or phylogenetic analyses, consistent with multiple relatively recent spillover events from domestic sheep to bighorn sheep rather than separate evolutionary origins in these two hosts [11].

We identified many genes that showed evidence for potential involvement in adaptation based on the detection of homologous recombination, which allows exchange of selectively advantageous genetic variation between bacterial cells [46,47]. Significant GO terms for these genes were related to the transport of organic substances including carbohydrates, the metabolism of which is a predicted virulence factor in *Mycoplasma* [70]. Furthermore, the significant GO terms included a specific group of transporters, the ATP-binding cassette (ABC) transporters, which are thought to play an important role in virulence for *Mycoplasma* and other bacterial species [95,97]. High homologous recombination in genes encoding transport proteins has also been observed across a wide range of bacterial species [46,98] and is thought to be driven by the direct contact of transport proteins with the extracellular environment, which could drive strong adaptive responses to environmental change.

We also identified genes for which presence or absence was associated with host species, which are candidate genes for putative differences in pathogenicity mechanisms resulting from evolutionary origins in separate host species or within-host habitats. Most of the genes identified as associated with host species were involved in carbon metabolism. For example, when comparing sheep versus goat clades, two annotated genes (*glpQ* and *rnhB*) were strongly associated with the goat clade, and both of these have functions related to the production of hydrogen peroxide, which is a byproduct of glycerol metabolism and considered to be a virulence factor for *Mycoplasma* [93]. The gene product of *glpQ*, which was present in all goat clade assemblies but only 22 % of sheep clade assemblies, performs enzymatic and regulatory roles in the catabolism of glycerol and its derivative glycerophosphocholine, which results in the production of hydrogen peroxide [70,92, 93]. The gene product of *rnhB*, which was present in 39 % of goat clade assemblies but no sheep clade assemblies, plays a role in DNA repair and has been shown to protect bacterial cells from oxidative damage in the presence of hydrogen peroxide [94].

When comparing caribou subclade assemblies to the rest of the sheep clade, 23 annotated genes were absent in the caribou assemblies but present in most or all other sheep clade genomes. One of these genes is a potential *Mycoplasma* virulence factor (*lppt*) that plays a role in adhesion to host cells [70]. The remaining 22 genes were enriched for biological processes and molecular functions involving catabolism of carbon sources other than glycerol, including genes encoding membrane transporters and catabolic enzymes, many of which showed evidence for homologous recombination in the analyses described earlier. These genes were involved in catabolism of sialic acid (*nanA*, *nanE*, *nagA*, *nagC* and *nagE*), N-acetylglucosamine (*nagA*, *nagC* and *nagE*), maltose and maltodextrin (*malC*, *malK*), fructose and mannose (*fruA*), trehalose (*treP*) and ascorbate (*ulaA*). Many of these genes have been proposed as virulence factors in *Mycoplasma* and other bacterial species, including *nanA* and *nanE* for *M. hyorhinis* and *M. alligatoris* [30,99]; *nagA*, *nagC* and *nagE* for *M. hyopneumoniae* and other bacterial species [30,100, 101]; *fruA* for *M. gallisepticum* [33,102]; *treP* for a wide range of bacterial species [103]; and *ulaA* for *M. hyopneumoniae* [104]. The absence of these genes in caribou assemblies suggests that these strains are more restricted in the types of carbon sources that can be utilized compared to other strains. These genes may have been lost over time through reductive evolution if certain carbon sources were absent or redundant in the environment [105] or through adaptive gene loss if the genes were disadvantageous in the environment [106]. The absence of these genes in Alaska wildlife strains could contribute to the apparently low virulence of these strains, potentially due to lower growth opportunities resulting from the inability to use available carbon sources, reduced production of toxic byproducts of carbon catabolism or other factors. However, a larger sample size of *M. ovipneumoniae* from Alaska wildlife would be needed to confirm that these genes are consistently absent across strains.

The genes absent in caribou assemblies also included *cas1* and *cas2*, which are both critical to the CRISPR-Cas system. Furthermore, other critical components of the CRISPR-Cas system showed evidence of being nonfunctional in these assemblies; the *cas9* gene was truncated, and the number of CRISPR repeats was lower than for most other genomes in our study. In contrast, the CRISPR-Cas system appears to be functional in most other strains in our study. CRISPR-Cas systems provide defence mechanisms for bacteria against foreign invaders such as phages and plasmids but also play a role in virulence through a wide range of mechanisms [107,109]. For example, the inactivation of *cas9* has been shown to decrease virulence for some bacteria by decreasing adhesion, toxin production and intracellular survival [108,110, 111]. Therefore, the absence of a functional CRISPR-Cas system in the caribou strains could be related to the apparently lower virulence of these strains.

Some of our tests regarding adaptation did not result in the identification of candidate genes. For example, we did not identify any genes that were significantly associated with domestic versus bighorn sheep hosts, and therefore, we did not identify any genes for which presence or absence was associated with spillover and persistence in bighorn sheep. However, larger sample sizes would be required to fully evaluate whether the presence or absence of certain genes may create an adaptive advantage in bighorn sheep. We also did not identify any core genes with evidence for positive or diversifying selection across our dataset based on *dN/dS* ratios. However, these analyses are only able to identify genes for which selection has caused nonsynonymous nucleotide changes in multiple aas and are unable to identify genes for which selection has acted on only a small portion of the gene, such as a single aa. Furthermore, these analyses focused only on core genes, which are predicted to experience strong purifying selection, whereas accessory genes may be more likely to be involved in niche specialization and experience positive or diversifying selection [90].

We also identified ten genes that are present across some or all *M. ovipneumoniae* strains in our dataset that have been implicated as potential virulence factors for *M. ovipneumoniae* and other *Mycoplasma* species in prior studies. These included additional genes involved in adhesion and glycerol metabolism and the associated production of hydrogen peroxide, as well as hemolysis and other functions.

### Phylogeography

We found high genetic diversity for *M. ovipneumoniae* and minimal phylogenetic clustering by geographic distance between herds, consistent with previous MLST studies in the USA and Europe [3,11, 24]. These patterns likely result from large population sizes and strong anthropogenic determinants of long-distance movement patterns for domestic sheep. However, all US assemblies, with the exception of the caribou assemblies, clustered into one highly supported clade with a deep ancestral node. This pattern suggests relatively low rates of new introductions of *M. ovipneumoniae* into the USA in recent history. The caribou strains clustered more closely with strains from France and China than from the USA but, nonetheless, were highly divergent from those strains, indicating that our dataset does not include any close relatives to the caribou strains. These results suggest that the caribou strains may be descendants of a separate introduction than the introductions from which all other US strains in our dataset are descended, potentially originating from a geographic region that was not sampled in our dataset. Although our dataset only includes three samples from caribou, the MLST sequences of these three samples are identical to all other *M. ovipneumoniae* from caribou and Dall’s sheep in Alaska sequenced over a 15-year period (2004–2019) [43]. This indicates that *M. ovipneumoniae* in Alaska wildlife populations may be characterized by low genomic diversity and that the three assemblies in our dataset may be representative for these populations, although more samples would be needed to confirm these patterns. The low MLST diversity of the caribou strains suggests that the introduction from which these strains descended may have been a relatively recent event. However, our analyses cannot identify the geographic source, date or number of *M. ovipneumoniae* introductions into Alaska wildlife populations. Furthermore, our analyses cannot identify whether the observed genomic differences for these strains originally evolved in caribou or Dall’s sheep populations, or another host species. To address these questions, more samples would be needed from more Alaska wildlife populations and all potential introduction source regions.

### Implications for pathogen monitoring

Genomic monitoring of *M. ovipneumoniae* is typically conducted by sequencing four MLST markers rather than whole genomes, due to the challenges and cost of culturing this pathogen and performing whole genome sequencing. One of the primary uses of MLST sequences has been the tracing of introduction sources for bighorn sheep populations. We found that strains with >99.9 % similarity in MLST sequences were also highly similar at a genome-wide level, suggesting that strains with highly similar MLST sequences are likely indicators of recent transmission events. We also found that measures of divergence between strains were overall similar between MLST sequences and whole-genome sequences, indicating that MLST sequences can be used as rough estimates of genome-wide divergence. However, MLST divergence estimates had greater variance and tended to underestimate genome-wide divergence, especially between sheep and goat strains. Phylogenies generated with concatenated MLST markers separated the sheep and goat clades with high bootstrap support, indicating that MLST markers can also be used to reliably distinguish these clades.

### Limitations

One limitation of this study was that many of the genes identified in the *M. ovipneumoniae* assemblies remained unannotated due to the current lack of genetic information for this non-model organism. Some of the genes identified as outliers in our analyses were not annotated, and therefore, the potential involvement of those genes in pathogenicity remains unexplored. Also, the gene clustering analysis performed by Roary appeared to separate some orthologous genes by clades or subclades due to the high genetic variation in our dataset, causing some orthologous genes that were shared across all assemblies to appear to be present in only one clade. To minimize the separation of orthologous genes, we reduced the Roary minimum per cent identity parameter to 92 %, with that value chosen based on ANI values (as described in the Methods section); reducing this value further ran the risk of grouping non-orthologous genes. To account for the potential impact of orthologous gene separation on comparisons of gene presence or absence across clades and host species, we used gene annotations rather than gene clusters to define a gene as present or absent in each assembly. Another limitation of this study was the relatively low sample sizes, especially for the caribou strains, for which only three samples were available that had been collected from the same herd within a 24-h period. MLST analyses indicate very low diversity across Alaska wildlife populations, indicating that these three assemblies may be representative of these populations, but future studies should use more samples to determine whether the patterns observed here are consistent across populations.

## Conclusions

In this study, we substantially increased the number of publicly available genome assemblies for *M. ovipneumoniae*, as well as the geographic and host species ranges of available assemblies, and provided new insight into phylogenetic structure and putative pathogenicity mechanisms for this bacterium. Phylogenetic analyses indicated two clades with species-level divergence that likely evolved separately in domestic sheep versus domestic goat hosts, and high diversity and minimal geographic clustering within those two clades. Comparative genomic analyses identified multiple genes and cellular processes that may be involved in pathogenicity mechanisms across clades and host species. In particular, we found evidence that carbon metabolism may play a role in virulence and adaptation to host immune defences for *M. ovipneumoniae*. The results of these analyses, along with the database of genome assemblies generated here, provide a resource for identifying genes and cellular processes to target for further investigation into the epidemiology and virulence of this pathogen.

## supplementary material

10.1099/mgen.0.001279 Uncited Supplementary Material 1.

10.1099/mgen.0.001279 Uncited Supplementary Material 2.

## Abbreviations

ABC: ATP-binding cassette
ANI: average nucleotide identity
ATCC: American Type Culture Collection
BAPS: Bayesian Analysis of Population Structure
CRISPR-Cas: clustered regularly interspaced short palindromic repeats and associated proteins
GO: gene ontology
gyrB: gyrase B
IGS: intergenic spacer region
MLST: multilocus sequence typing
NCBI: National Center for Biotechnology Information
ONT: Oxford Nanopore Technologies
PacBio: Pacific Biosciences
pan-GWAS: pangenome-wide association study
qPCR: quantitative PCR
rpoB: RNA polymerase B
VFDB: virulence factor database

## References

[1] Balish M, Bertaccini A, Blanchard A, Brown D, Browning G (2019). Recommended rejection of the names *malacoplasma* gen. nov., *mesomycoplasma* gen. nov., *metamycoplasma* gen. nov., *metamycoplasmataceae* fam. nov., *mycoplasmoidaceae* fam. nov., *mycoplasmoidales* ord. nov., *mycoplasmoides* gen. nov., *mycoplasmopsis* gen. nov. [gupta, sawnani, adeolu, alnajar and oren 2018] and all proposed species comb. nov. placed therein. Int J Syst Evol Microbiol.

[2] Gupta RS, Son J, Oren A (2019). A phylogenomic and molecular markers based taxonomic framework for members of the order *Entomoplasmatales*: proposal for an emended order *Mycoplasmatales* containing the family *Spiroplasmataceae* and emended family *Mycoplasmataceae* comprised of six genera. Antonie van Leeuwenhoek.

[3] Jaÿ M, Ambroset C, Tricot A, Colin A, Tardy F (2020). Population structure and antimicrobial susceptibility of *Mycoplasma ovipneumoniae* isolates in France. Vet Microbiol.

[4] Nicholas RAJ, Ayling RD, Loria GR (2008). Ovine mycoplasmal infections. Small Ruminant Res.

[5] Maksimović Z, Rifatbegović M, Loria GR, Nicholas RAJ (2022). *Mycoplasma ovipneumoniae*: a most variable pathogen. Pathogens.

[6] Manlove K, Branan M, Baker K, Bradway D, Cassirer EF (2019). Risk factors and productivity losses associated with *Mycoplasma ovipneumoniae i*nfection in United States domestic sheep operations. Prev Vet Med.

[7] Besser TE, Levy J, Ackerman M, Nelson D, Manlove K (2019). A pilot study of the effects of *Mycoplasma ovipneumoniae* exposure on domestic lamb growth and performance. PLoS One.

[8] DaMassa AJ, Wakenell PS, Brooks DL (1992). Mycoplasmas of goats and sheep. J Vet Diagn Invest.

[9] Alley MR, Ionas G, Clarke JK (1999). Chronic non-progressive pneumonia of sheep in New Zealand - a review of the role of *Mycoplasma ovipneumoniae*. N Z Vet J.

[10] Besser TE, Cassirer EF, Yamada C, Potter KA, Herndon C (2012). Survival of bighorn sheep (*Ovis canadensis*) commingled with domestic sheep (*Ovis aries*) in the absence of *Mycoplasma ovipneumoniae*. J Wildl Dis.

[11] Kamath PL, Manlove K, Cassirer EF, Cross PC, Besser TE (2019). Genetic structure of *Mycoplasma ovipneumoniae* informs pathogen spillover dynamics between domestic and wild Caprinae in the western United States. Sci Rep.

[12] Handeland K, Tengs T, Kokotovic B, Vikøren T, Ayling RD (2014). *Mycoplasma ovipneumoniae* - a primary cause of severe pneumonia epizootics in the Norwegian Muskox (*Ovibos moschatus*) population. PLoS One.

[13] Besser TE, Highland M, Baker K, Cassirer EF, Anderson NJ (2012). Causes of pneumonia epizootics among bighorn sheep, Western United States, 2008–2010. Emerg Infect Dis.

[14] Black SR, Barker IK, Mehren KG, Crawshaw GJ, Rosendal S (1988). An epizootic of *Mycoplasma ovipneumoniae* infection in captive Dall’s sheep (*Ovis dalli dalli*). J Wildl Dis.

[15] Cassirer EF, Manlove KR, Almberg ES, Kamath PL, Cox M (2018). Pneumonia in bighorn sheep: risk and resilience. J Wildl Manag.

[16] Manlove K, Cassirer EF, Cross PC, Plowright RK, Hudson PJ (2016). Disease introduction is associated with a phase transition in bighorn sheep demographics. Ecology.

[17] Plowright RK, Manlove KR, Besser TE, Páez DJ, Andrews KR (2017). Age-specific infectious period shapes dynamics of pneumonia in bighorn sheep. Ecol Lett.

[18] Garwood TJ, Lehman CP, Walsh DP, Cassirer EF, Besser TE (2020). Removal of chronic *Mycoplasma ovipneumoniae* carrier ewes eliminates pneumonia in a bighorn sheep population. Ecol Evol.

[19] Johnson BM, Stroud-Settles J, Roug A, Manlove K (2022). Disease ecology of a low-virulence *Mycoplasma ovipneumoniae* strain in a free-ranging desert bighorn sheep population. Animals.

[20] Walsh DP, Felts BL, Cassirer EF, Besser TE, Jenks JA (2023). Host vs. pathogen evolutionary arms race: effects of exposure history on individual response to a genetically diverse pathogen. Front Ecol Evol.

[21] Besser TE, Cassirer EF, Lisk A, Nelson D, Manlove KR (2021). Natural history of a bighorn sheep pneumonia epizootic: source of infection, course of disease, and pathogen clearance. Ecol Evol.

[22] Niang M, Rosenbusch RF, DeBey MC, Niyo Y, Andrews JJ (1998). Field isolates of *Mycoplasma ovipneumoniae* exhibit distinct cytopathic effects in ovine tracheal organ cultures. J Vet Med Ser A.

[23] Yang FL, Tang C, Wang Y, Zhang HR, Yue H (2011). Genome sequence of *Mycoplasma ovipneumoniae s*train SC01. J Bacteriol.

[24] Einarsdottir T, Gunnarsson E, Hjartardottir S (2018). Icelandic ovine *Mycoplasma ovipneumoniae* are variable bacteria that induce limited immune responses *in vitro* and *in vivo*. J Med Microbiol.

[25] Dudek K, Sevimli U, Migliore S, Jafarizadeh A, Loria GR (2022). Vaccines for Mycoplasma diseases of small ruminants: a neglected area of research. Pathogens.

[26] Jiang Z, Song F, Li Y, Xue D, Zhao N (2017). Capsular polysaccharide of *Mycoplasma ovipneumoniae* induces sheep airway epithelial cell apoptosis via ROS-dependent JNK/P38 MAPK pathways.. Oxid Med Cell Longev.

[27] Jiang ZJ, Song FY, Li YN, Xue D, Deng GC (2017). Capsular polysaccharide is a main component of *Mycoplasma ovipneumoniae* in the pathogen-induced toll-like receptor-mediated inflammatory responses in sheep airway epithelial cells. Mediators Inflamm.

[28] Li Y, Wang R, Sun W, Song Z, Bai F (2020). Comparative genomics analysis of *Mycoplasma capricolum* subsp. *capripneumoniae* 87001. Genomics.

[29] Chen S, Hao H, Zhao P, Liu Y, Chu Y (2018). Genome-wide analysis of *Mycoplasma bovirhinis* GS01 reveals potential virulence factors and phylogenetic relationships. G3.

[30] Trueeb BS, Gerber S, Maes D, Gharib WH, Kuhnert P (2019). Tn-sequencing of *Mycoplasma hyopneumoniae* and *Mycoplasma hyorhinis mutant* libraries reveals non-essential genes of porcine mycoplasmas differing in pathogenicity. Vet Res.

[31] Brown DR, Farmerie WG, May M, Benders GA, Durkin AS (2011). Genome sequences of *Mycoplasma alligatoris* A21JP2T and *Mycoplasma crocodyli* MP145T. J Bacteriol.

[32] Liu W, Xiao S, Li M, Guo S, Li S (2013). Comparative genomic analyses of *Mycoplasma hyopneumoniae* pathogenic 168 strain and its high-passaged attenuated strain. BMC Genomics.

[33] Szczepanek SM, Tulman ER, Gorton TS, Liao X, Lu Z (2010). Comparative genomic analyses of attenuated strains of *Mycoplasma gallisepticum*. Infect Immun.

[34] Sheppard SK, Guttman DS, Fitzgerald JR (2018). Population genomics of bacterial host adaptation. Nat Rev Genet.

[35] Keen EC, Choi J, Wallace MA, Azar M, Mejia-Chew CR (2021). Comparative genomics of *Mycobacterium avium* complex reveals signatures of environment-specific adaptation and community acquisition. mSystems.

[36] Wang J, Li Y, Pinto-Tomás AA, Cheng K, Huang Y (2022). Habitat adaptation drives speciation of a *Streptomyces* species with distinct habitats and disparate geographic origins. mBio.

[37] Maksimović Z, De la Fe C, Amores J, Gómez-Martín Á, Rifatbegović M (2017). Comparison of phenotypic and genotypic profiles among caprine and ovine *Mycoplasma ovipneumoniae* strains. Vet Rec.

[38] Parham K, Churchward CP, McAuliffe L, Nicholas RAJ, Ayling RD (2006). A high level of strain variation within the *Mycoplasma ovipneumoniae* population of the UK has implications for disease diagnosis and management. Vet Microbiol.

[39] Cassirer EF, Manlove KR, Plowright RK, Besser TE (2017). Evidence for strain‐specific immunity to pneumonia in bighorn sheep. J Wildl Manag.

[40] Besser TE, Cassirer EF, Potter KA, Foreyt WJ (2017). Exposure of bighorn sheep to domestic goats colonized with *Mycoplasma ovipneumoniae* induces sub-lethal pneumonia. PLoS One.

[41] Foreyt WJ, Jenkins EJ, Appleyard GD (2009). Transmission of lungworms (*Muellerius capillaris*) from domestic goats to bighorn sheep on common pasture. J Wildl Dis.

[42] Wehausen JD (2011). Domestic sheep, bighorn sheep, and respiratory disease: a review of the experimental evidence. California Fish Game.

[43] Lieske CL, Gerlach R, Francis M, Beckmen KB (2022). Multilocus sequence typing of *Mycoplasma ovipneumoniae* detected in Dall’s sheep (*Ovis dalli dalli*) and Caribou (*Rangifer tarandus grantii*) in Alaska, USA. J Wildl Dis.

[44] May M, Brown DR (2009). Diversifying and stabilizing selection of sialidase and N-acetylneuraminate catabolism in *Mycoplasma synoviae*. J Bacteriol.

[45] Cao P, Guo D, Liu J, Jiang Q, Xu Z (2017). Genome-wide analyses reveal genes subject to positive selection in *Pasteurella multocida*. Front Microbiol.

[46] González-Torres P, Rodríguez-Mateos F, Antón J, Gabaldón T (2019). Impact of homologous recombination on the evolution of prokaryotic core genomes. mBio.

[47] Arnold BJ, Huang IT, Hanage WP (2022). Horizontal gene transfer and adaptive evolution in bacteria. Nat Rev Microbiol.

[48] Cottew GS (1979). The Mycoplasmas, Vol II, Human and Animal Mycoplasmas.

[49] Carmichael LE, St George TD, Sullivan ND, Horsfall N (1972). Isolation, propagation, and characterization studies of an ovine *Mycoplasma* responsible for proliferative interstitial pneumonia. Cornell Vet.

[50] Jennings-Gaines JE, Edwards WH, Wood ME, Fox KA, Wolfe LL (2016). An improved method for culturing *Mycoplasma ovipneumoniae* from field samples. Biennial Symposium of the Northern Wild Sheep and Goat Council.

[51] Kim D, Song L, Breitwieser FP, Salzberg SL (2016). Centrifuge: rapid and sensitive classification of metagenomic sequences. Genom Res.

[52] Wick RR, Judd LM, Cerdeira LT, Hawkey J, Méric G (2021). Trycycler: consensus long-read assemblies for bacterial genomes. Genome Biol.

[53] Walker BJ, Abeel T, Shea T, Priest M, Abouelliel A (2014). Pilon: an integrated tool for comprehensive microbial variant detection and genome assembly improvement. PLoS One.

[54] Bankevich A, Nurk S, Antipov D, Gurevich AA, Dvorkin M (2012). SPAdes: a new genome assembly algorithm and its applications to single-cell sequencing. J Comput Biol.

[55] Parks DH, Imelfort M, Skennerton CT, Hugenholtz P, Tyson GW (2015). CheckM: assessing the quality of microbial genomes recovered from isolates, single cells, and metagenomes. Genome Res.

[56] Jain C, Rodriguez-R LM, Phillippy AM, Konstantinidis KT, Aluru S (2018). High throughput ANI analysis of 90K prokaryotic genomes reveals clear species boundaries. Nat Commun.

[57] Seemann T (2014). Prokka: rapid prokaryotic genome annotation. Bioinformatics.

[58] Hyatt D, Chen G-L, Locascio PF, Land ML, Larimer FW (2010). Prodigal: prokaryotic gene recognition and translation initiation site identification. BMC Bioinform.

[59] Camacho C, Coulouris G, Avagyan V, Ma N, Papadopoulos J (2009). BLAST+: architecture and applications. BMC Bioinform.

[60] Finn RD, Clements J, Eddy SR (2011). HMMER web server: interactive sequence similarity searching. Nucleic Acids Res.

[61] Page AJ, Cummins CA, Hunt M, Wong VK, Reuter S (2015). Roary: rapid large-scale prokaryote pan genome analysis. Bioinformatics.

[62] Löytynoja A, Russell D (2014). Multiple Sequence Alignment Methods Methods in Molecular Biology (Methods and Protocols.

[63] Cantalapiedra CP, Hernández-Plaza A, Letunic I, Bork P, Huerta-Cepas J (2021). eggNOG-mapper v2: functional annotation, orthology assignments, and domain prediction at the metagenomic scale. Mol Biol Evol.

[64] Huerta-Cepas J, Szklarczyk D, Heller D, Hernández-Plaza A, Forslund SK (2019). eggNOG 5.0: a hierarchical, functionally and phylogenetically annotated orthology resource based on 5090 organisms and 2502 viruses. Nucleic Acids Res.

[65] Ruiz-Perez CA, Conrad RE, Konstantinidis KT (2021). MicrobeAnnotator: a user-friendly, comprehensive functional annotation pipeline for microbial genomes. BMC Bioinform.

[66] Gaeta NC, de Sá Guimarães AM, Timenetsky J, Clouser S, Gregory L (2022). The first *Mycoplasma ovipneumoniae* recovered from a sheep with respiratory disease in Brazil - draft genome and genomic analysis. Vet Res Commun.

[67] Buchfink B, Reuter K, Drost H-G (2021). Sensitive protein alignments at tree-of-life scale using DIAMOND. Nat Methods.

[68] Liu B, Zheng D, Jin Q, Chen L, Yang J (2018). VFDB 2019: a comparative pathogenomic platform with an interactive web interface. Nucleic Acids Res.

[69] Chen L, Yang J, Yu J, Yao Z, Sun L (2005). VFDB: a reference database for bacterial virulence factors. Nucleic Acids Res.

[70] Yiwen C, Yueyue W, Lianmei Q, Cuiming Z, Xiaoxing Y (2021). Infection strategies of mycoplasmas: unraveling the panoply of virulence factors. Virulence.

[71] Kozlov AM, Darriba D, Flouri T, Morel B, Stamatakis A (2019). RAxML-NG: a fast, scalable and user-friendly tool for maximum likelihood phylogenetic inference. Bioinformatics.

[72] Perron GG, Lee AEG, Wang Y, Huang WE, Barraclough TG (2012). Bacterial recombination promotes the evolution of multi-drug-resistance in functionally diverse populations. Proc R Soc B.

[73] Cavassim MIA, Andersen SU, Bataillon T, Schierup MH (2021). Recombination facilitates adaptive evolution in rhizobial soil bacteria. Mol Biol Evol.

[74] Hsieh Y-C, Li S-W, Chen Y-Y, Kuo C-C, Chen Y-C (2022). Global genome diversity and recombination in *Mycoplasma pneumoniae*. Emerg Infect Dis.

[75] Mostowy R, Croucher NJ, Andam CP, Corander J, Hanage WP (2017). Efficient inference of recent and ancestral recombination within bacterial populations. Mol Biol Evol.

[76] Corander J, Waldmann P, Sillanpää MJ (2003). Bayesian analysis of genetic differentiation between populations. Genetics.

[77] Yang ZH (2007). PAML 4: phylogenetic analysis by maximum likelihood. Mol Biol Evol.

[78] Melton AE, Chen S, Zhao Y, Fu C, Xiang Q-YJ (2020). Genetic insights into the evolution of genera with the eastern Asia-eastern North America floristic disjunction: a transcriptomics analysis. Am J Bot.

[79] Martinez-Gutierrez CA, Aylward FO (2019). Strong purifying selection is associated with genome streamlining in epipelagic Marinimicrobia. Genome Biol Evol.

[80] Brynildsrud O, Bohlin J, Scheffer L, Eldholm V (2016). Rapid scoring of genes in microbial pan-genome-wide association studies with Scoary. Genome Biol.

[81] San JE, Baichoo S, Kanzi A, Moosa Y, Lessells R (2019). Current affairs of microbial genome-wide association studies: approaches, bottlenecks and analytical pitfalls. Front Microbiol.

[82] Couvin D, Bernheim A, Toffano-Nioche C, Touchon M, Michalik J (2018). CRISPRCasFinder, an update of CRISRFinder, includes a portable version, enhanced performance and integrates search for Cas proteins. Nucleic Acids Res.

[83] Abby SS, Néron B, Ménager H, Touchon M, Rocha EPC (2014). MacSyFinder: a program to mine genomes for molecular systems with an application to CRISPR-Cas systems. PLoS One.

[84] Grissa I, Vergnaud G, Pourcel C (2007). CRISPRFinder: a web tool to identify clustered regularly interspaced short palindromic repeats. Nucleic Acids Res.

[85] Mizuki H, Shimoyama Y, Ishikawa T, Sasaki M (2022). A genomic sequence of the type II-A clustered regularly interspaced short palindromic repeats (CRISPR)/CRISPR-associated system in *Mycoplasma salivarium* strain ATCC 29803. J Oral Microbiol.

[86] Alexa A, Rahnenführer J (2022). TopGO: enrichment analysis for gene ontology. R package version 2.50.0.

[87] UniProt C (2023). UniProt: the universal protein knowledgebase in 2023. Nucleic Acids Res.

[88] Altschul SF, Gish W, Miller W, Myers EW, Lipman DJ (1990). Basic local alignment search tool. J Mol Biol.

[89] Paradis E, Schliep K (2019). ape 5.0: an environment for modern phylogenetics and evolutionary analyses in R. Bioinformatics.

[90] Preska Steinberg A, Lin M, Kussell E (2022). Core genes can have higher recombination rates than accessory genes within global microbial populations. eLife.

[91] McInerney JO, McNally A, O’Connell MJ (2017). Why prokaryotes have pangenomes. Nat Microbiol.

[92] Schmidl SR, Otto A, Lluch-Senar M, Piñol J, Busse J (2011). A trigger enzyme in *Mycoplasma pneumoniae*: impact of the glycerophosphodiesterase GlpQ on virulence and gene expression. PLoS Pathog.

[93] Blötz C, Stülke J (2017). Glycerol metabolism and its implication in virulence in *Mycoplasma*. FEMS Microbiol Rev.

[94] Gupta R, Chatterjee D, Glickman MS, Shuman S (2016). Division of labor among *Mycobacterium smegmatis* RNase H enzymes: RNase H1 activity of RnhA or RnhC is essential for growth whereas RnhB and RnhA guard against killing by hydrogen peroxide in stationary phase. Nucleic Acids Res.

[95] Zhu L, Shahid MA, Markham J, Browning GF, Noormohammadi AH (2019). Comparative genomic analyses of *Mycoplasma synoviae* vaccine strain MS-H and its wild-type parent strain 86079/7NS: implications for the identification of virulence factors and applications in diagnosis of *M. synoviae*. Avian Pathol.

[96] Akhtar AA, Turner DPJ (2022). The role of bacterial ATP-binding cassette (ABC) transporters in pathogenesis and virulence: therapeutic and vaccine potential. Microb Pathog.

[97] Sun Y, Luo HW (2018). Homologous recombination in core genomes facilitates marine bacterial adaptation. Appl Environ Microbiol.

[98] Yahara K, Didelot X, Jolley KA, Kobayashi I, Maiden MCJ (2016). The landscape of realized homologous recombination in pathogenic bacteria. Mol Biol Evol.

[99] Michaels DL, Moneypenny CG, Shama SM, Leibowitz JA, May MA (2019). Sialidase and N-acetylneuraminate catabolism in nutrition of *Mycoplasma alligatoris*. Microbiology.

[100] Almagro-Moreno S, Boyd EF (2009). Insights into the evolution of sialic acid catabolism among bacteria. BMC Evol Biol.

[101] Ansari S, Kumar V, Bhatt DN, Irfan M, Datta A (2022). N-acetylglucosamine sensing and metabolic engineering for attenuating human and plant pathogens. Bioengineering.

[102] Bekő K, Kovács ÁB, Kreizinger Z, Marton S, Bányai K (2020). Development of mismatch amplification mutation assay for the rapid differentiation of *Mycoplasma gallisepticum* K vaccine strain from field isolates. Avian Pathol.

[103] Vanaporn M, Titball RW (2020). Trehalose and bacterial virulence. Virulence.

[104] Li S, Fang L, Liu W, Song T, Zhao F (2019). Quantitative proteomic analyses of a pathogenic strain and Its highly passaged attenuated strain of *Mycoplasma hyopneumoniae*. Biomed Res Int.

[105] Moran NA (2002). Microbial minimalism: genome reduction in bacterial pathogens. Cell.

[106] Albalat R, Cañestro C (2016). Evolution by gene loss. Nat Rev Genet.

[107] Louwen R, Staals RHJ, Endtz HP, van Baarlen P, van der Oost J (2014). The role of CRISPR-Cas systems in virulence of pathogenic bacteria. Microbiol Mol Biol Rev.

[108] Wu Q, Cui L, Liu Y, Li R, Dai M (2022). CRISPR-Cas systems target endogenous genes to impact bacterial physiology and alter mammalian immune responses. *Mol Biomed*.

[109] Newsom S, Parameshwaran HP, Martin L, Rajan R (2021). The CRISPR-Cas mechanism for adaptive immunity and alternate bacterial functions fuels diverse biotechnologies. Front Cell Infect Microbiol.

[110] Sampson TR, Saroj SD, Llewellyn AC, Tzeng Y-L, Weiss DS (2013). A CRISPR/Cas system mediates bacterial innate immune evasion and virulence. Nature.

[111] Ma K, Cao Q, Luo S, Wang Z, Liu G (2018). *cas9* enhances bacterial virulence by repressing the regR transcriptional regulator in *Streptococcus agalactiae*. Infect Immun.

